# The Integration of Islamic Psychology with Acceptance and Commitment Therapy (ACT)

**DOI:** 10.1007/s11013-025-09924-5

**Published:** 2025-07-18

**Authors:** Imran Khan, Shaystah Dean, Damien Ridge, Nikolaos Souvlakis

**Affiliations:** 1 Barts and the London Centre for Primary Care, Wolfson Institute of Population Health, Queen Mary University of London, London, UK; 2 Department of Psychological Medicine, University of Otago, Christchurch, New Zealand; 3 Centre for Psychological Sciences, University of Westminster, New Cavendish St, London, UK

**Keywords:** Acceptance and commitment therapy (ACT), Islamic Psychology, Inequalities, Mental health, Muslims, NHS

## Abstract

Black and ethnic minority groups (diverse groups) face inequalities when it comes to healthcare in the United Kingdom, including access, stigma, and discrimination. Many of these diverse communities include Muslims. It is also known that barriers for Muslims especially involve accessing mental health services because of fears of stereotyping, stigma, and expected NHS incongruence with religious beliefs. Many Muslims make connections between religious attributions and mental health issues and consider religion a source of support. There have been efforts to construct an Islamic model of the self as a framework for healing in the therapeutic context for Muslim patients. This article develops an initial framework to integrate an Islamic model of the self with acceptance and commitment therapy (ACT). We will detail the rationale for this spiritual integration in the development of a potential mental health intervention for Muslim patients. We describe how ACT is useful, given the overlap of ACT’s aim to increase psychological flexibility with the therapeutic and spiritual goals of Islamic Psychology. We use a case example to briefly illustrate the points of congruence between ACT and Islamic principles. This approach has the potential to enhance access to healthcare for Muslim patients via the NHS. Further work will be needed to develop a practical tool for therapists who wish to deliver an Islamic based therapy for Muslims using ACT as a framework.

## Introduction

Western mental healthcare often treats neuropsychiatric knowledge as superior, which can lead to the marginalisation of racially minoritised people whose beliefs are seen as less valid, and even as barriers to well-being (Fletcher, 2021). This has the potential to alienate diverse groups (who feel they cannot bring their whole selves into NHS therapies), and contribute to the inequality that we see in accessing mental health care services, where the uptake of these services is low among many minority groups (NHS, 2023; Prajapati & Liebling, 2022).

The evidence shows that religious patients can interpret their illnesses and their healing in helpful and meaningful ways within the cosmology of their own belief system (Ridge et al., 2023). However, their approaches may not align with secular western models of illness, leading to an “epistemic tension” between seemingly opposing approaches to mental health (Bansal et al., 2022, p. 20). A recent meta-ethnography outlines how such tensions can create barriers for diverse groups in accessing mental health care provision, including needing to manage knowledge deficits of professionals, creating distance with care, and exacerbating inequalities (Bansal et al., 2022).

One in three ethnic minorities in the UK are Muslim (MCB, 2020). This means that Muslims as a whole are disproportionately affected by inequalities that exist in mental health care provision in the UK (Prajapati & Liebling, 2022). According to the recent census data, 40% of Muslims live in the most deprived local authority districts (MCB, 2022). In addition, the 3.9 million (and rising) Muslims in the UK have specific and rarely acknowledged needs including unique traumas, including the recent rise of anti-Muslim hatred (Bansal et al., 2022; Dearden, 2025; Mohdin & Osuh, 2025). Therefore, attempts to address barriers to the uptake of mental health services by Muslims to improve equality of access are long overdue. One common reported barrier is questioning whether the therapist or practitioner will recognise and work with Muslim patient attributions of mental health (whether useful or unhelpful) related to religion (Islam et al., 2015). In addition, fear of anti-Muslim sentiment and negative community stereotyping of services mean that the perceived “costs of help-seeking could easily outweigh the opportunities offered by seeking respite from the symptoms of poor mental health” (Bansal et al., 2022, p. 22; Weatherhead & Daiches, 2010). In Tower Hamlets, NHS Talking Therapies data show that English-speaking Bangladeshi (predominantly Muslim) patients have a lower recovery rate (38%) compared to White British patients (58%) (NHS, 2024). Addressing these barriers is therefore a critical public health concern and crucial to prevent further alienation and ongoing distrust in public health institutions.

Religious beliefs are frequently sidelined in healthcare in a way that may marginalise Muslim users of mental health services (Alam, 2023). A recent large survey of minoritised ethnic men in two London boroughs, carried out by Social Action for Health, found that “religion and spiritual beliefs cannot be separated from the conversation around minoritised ethnic men's mental health” (SAfH, 2024, p. 3). In a national survey of Muslims, of the 332 respondents surveyed, 84% expressed a desire for faith informed counselling (Shaikh & Chowdhury, 2021). Intersectionally, dimensions like race, culture, and ethnicity are the focus of addressing health inequalities. Religious beliefs feature less often as an included dimension when considering barriers to mental health support (Bansal et al., 2022). Nevertheless, an intervention addressing such barriers could have broader applicability than a cultural intervention for a specific community (e.g. the Pakistani or Bangladeshi community). Therefore, religion seen in this way is a particularly salient consideration to target in intervention development.

Inequalities have been recognised with attempts to make interventions more culturally sensitive (Ridge et al., 2023). There have also been attempts to adapt Western-based interventions like cognitive behavioural therapy (CBT) to work better with the value systems of Muslim patients (Abrar & Hargreaves, 2023; Cucchi, 2022). However, some have viewed this approach as a kind of “Islamisation” (Rothman, 2018), where adaptions are fundamentally secular models but interjected with examples from the Islamic tradition in areas of congruence. Here, secular mechanisms towards recovery are identified in advance and become the primary focus (Haque et al., 2016). These kinds of adaptations may not be enough when dealing with issues important to Muslim patients, like connecting with *Allah* (God), dealing with conflicting values, or experiencing demonic whisperings—subtle, harmful thoughts linked to evil. As a result, such adaptations may overlook Islamic approaches to addressing these concerns.

Many practitioners and researchers have tried to engage with religious patients via a sense of curiosity around their beliefs, in a similar spirit to how anthropology might engage with non-Western cultures (Bhui et al., 2021). These approaches, although well intentioned, can suggest that there is no need to further examine religious or spiritual problems in research, even though they are signs of a deeper distress that represents an appropriate target for research and treatment (Pargament, 2011). Although curiosity alone can work with and recognise patients’ beliefs, it may be restricted in investigating treatment approaches that align to the overall tradition that the patient belongs to.

There have been psycho-therapeutic models developed that are regarded as ontologically rooted in the Islamic tradition that could provide an important new dimension to adapted interventions (Haque et al., 2016; Kaplick & Skinner, 2017; Keshavarzi et al., 2020; Maynard & Dharamsi, 2019; Rothman, 2021). These models may address some reported concerns by Muslim service users around a secular approach to therapy. However, there is a dearth of literature on the integration of these approaches into the care of Muslims. How are these approaches best delivered? What is the connection with Western evidence-based therapeutic modalities? Can approaches in Islamic Psychology accommodate an evidence-based therapy without it being dismissed as an “Islamisation” of a particular modality? Are there any other approaches that could work better?

We argue for the need to investigate a contemporary, evidence-based mode of therapy that aligns with the aims and ontology of Islam. We propose that acceptance and commitment therapy (ACT) has potential in this area. ACT is transdiagnostic and has been shown to correlate to positive well-being outcomes following depression, anxiety and some forms of psychosis (Hayes, 2016; Hayes & Smith, 2005; Hooper & Larsson, 2015). ACT also promotes psychological flexibility, which may offer an ontological bridge to the core Islamic concepts of patience and submission in the face of suffering (Tan, 2016). It has the potential to provide a framework for faith-centred interventions for Muslims by integrating Islamic models with psychotherapy—particularly in contexts where well-developed, deliverable Islamic therapeutic approaches are limited.

To address inequalities in accessing mental healthcare services, such an intervention could be made available via the NHS if demonstrated to improve mental well-being. It has been reported in a recent survey that many Muslims do access mental health services but wanted faith to inform their counselling-despite viewing practitioners in mainstream settings as better qualified than those in faith only based settings (Shaikh & Chowdhury, 2021). In addition, given the high proportion of Muslims living in deprived areas, there is a need to address financial barriers associated in accessing therapy (Shaikh & Chowdhury, 2021). This highlights the urgency of developing a faith-centred mental health counselling service suitable to Muslims within the current NHS talking therapies infrastructure. Such an approach could pave the way for targeted mental health interventions for other under-served groups with diverse beliefs to be made available via the NHS.

Accepting alternative approaches to mental healthcare can be challenging; however, such resistance is increasingly being questioned. The “decolonising healthcare” movement advocates for the recognition and integration of non-Western healing approaches, and challenges the dominance of Western medicine and psychology (Lokugamage et al., 2020; Quijano, 2007). It calls for cultural and epistemological humility in mental health services, encouraging professionals to acknowledge diverse worldviews—including religious or indigenous perspectives on well-being—rather than solely relying on biological or psychological explanations. This shift aligns with broader trends in psychiatry and psychology that emphasise integrated, relational, and embodied understandings of health, paving the way for a more inclusive approach that values alternative paradigms in patient care, like Islamic Psychology (Curtis et al., 2019; Khan & Saad, 2022; Siegel & Drulis, 2023; Tynan, 2021). For example, as we will discuss in this paper, traditional Islamic Psychology or (*ilm-al-nafs*) recognises a soul as a metaphysical entity, along with its purification and relationship with the heart, mind, and surroundings. This creates a framework for recovery and a path towards healing involving Divine proximity (Rothman, 2021). As a metaphor, this recognition could be offered to Muslim patients by practitioners of any or no religious background, and in ways which support mental health.

## What is an Islamic Psychology?

Islamic Psychology is an approach to mental health that integrates Islamic beliefs and metaphysical concepts into well-being, rather than simply attributing Western models (Rothman, 2018). Unlike conventional therapies rooted in secular notions of the self, Islamic Psychology acknowledges the spiritual core of a person, including the soul, divine connection, and influences such as temperaments and the role of the demonic (Skinner, 2010). By incorporating these elements, it potentially provides a more holistic healing process that alligns with Muslim beliefs, addressing concerns about the suitability of some mainstream therapies for Muslim patients.

In recent years, there have been important contributions made in developing Islamic psychotherapy—an approach that applies Islamic Psychology, and is grounded in normative Islamic concepts of the self (Rothman & Coyle, 2018). These approaches, referred to as “bottom up”, recognise that the heart is the centre of perception and thought. Islamic approaches focus on four elements that relate to the theological composition of the self: the *nafs* (lower self), *qalb* (heart), ‘*aql* (intellect), and *Ruh* (the Divine Spirit) (Rothman & Coyle, 2018). The aim is to ensure the heart moves towards a *fitra* state (i.e. a state that in congruent with ones being) that harmonises with the Divine element known as the *Ruh*. It represents is a move away from the ephemeral and is said to lead to a contented soul that is in submission to *Allah*. This transition is articulated in the Qur’an by the following verse: “[But] you, soul at peace, return to your Lord well pleased and well pleasing” (Haleem, 2005, pp. 89, 26–27). The efforts to move the heart in such directions can include avoidance of ‘vices’ and committing to ‘virtuous’ behaviours, as defined by the ethics of the Islamic faith. It also involves understanding the workings of the *nafs* (the lower self or base desires) and learning to manage them, much like a rider controls their horse. The ‘*aql* (intellect) represents the rider, while the *nafs* represents the horse, which must be reined in. Through discipline, the *nafs* can be brought to a regulated state. The *nafs* through discipline can be contented. This framework encompasses the Islamic revelatory sources in understanding the self and healing (Rothman, 2021).

Another approach that incorporates the whole Islamic self, is known as Traditional Islamic Integrated Therapy (TIIP). TIPP recognises healing and well-being is holistic, with the ultimate aim of achieving a sound heart (Keshavarzi et al., 2020). Such approaches to integrating the self may potentially address the needs for Muslim’s who believe available talking therapies do not fit their core beliefs. We elaborate further on these aspects of the self later in this paper.

As these encouraging approaches to Islamic psychotherapy have recently been developed, we must ask about how these approaches can be used in clinical practice? The TIIP approach suggests theological and philosophical ways of integration of existing therapies like CBT, to be used in conjunction with the wider framework of an Islamic Psychology (Keshavarzi et al., 2020). For example, as the ‘*aql* (intellect) plays a significant role in controlling the *nafs* (lower self), CBT being an approach that targets unhelpful thought patterns through cognitive effort can be seen as similar in terms of process (Haque et al., 2016).

However, we believe it is also meaningful, and considered fundamental to Islamic ontology, to recognise the need in Muslims to (re)connect to the Divine. Connecting to the Divine from an Islamic perspective can mean being in complete submission to *Allah*’s Will, while committing to actions to draw closer to *Allah*. Here, we argue that acceptance and commitment therapy (ACT), as an established talking therapy, may be a suitable vehicle for integrating Islamic ontology through acceptance of things one can’t change, in addition to its other psychological benefit, of integrating values, such as those to do with the Islamic self. The approach articulated in this paper seeks to use the Islamic self as articulated by Rothman and Coyle (2018) as a foundation to engage with the entire self, which includes metaphysical entities like the spiritual heart and spirit, while recognising the importance to acknowledge the epistemology of evidence-based practice (Fadl, 2015).

## Acceptance and Commitment Therapy (ACT) and Integration with Islamic Ontology

Acceptance and commitment therapy (ACT) was developed by US Psychologist Steven Hayes and colleagues and empirically supported as a useful model for positive change in people’s lives (Hayes, 2004; Hooper & Larsson, 2015). ACT provides a modality that can change a person’s relationship to their thoughts, using a language that is accessible, while resonating with the wisdoms of ancient traditions. Thus, ACT allows us to focus on a new way to integrate spiritual care, with respect to an Islamic Psychology by making us aware of the fundamental notions of suffering, acceptance, and meaning.

In traditional CBT, there is no explicit consideration of psychological pain in terms of spiritual growth. Anxiety disorders are viewed as arising from faulty cognitions and therefore treated by altering the content of these cognitions (Arch et al., 2012; Tan, 2016). There are adaptations to CBT such as mindfulness-based cognitive therapy (MBCT), which integrate elements that acknowledge the transformative potential of mindfulness (Fennell & Segal, 2013). ACT is arguably better at helping people live with painful or uncomfortable experiences (Harris, 2022; Ruiz, 2012). ACT doesn’t fight pain; instead, it teaches people to open up to pain, defuse from it, and still commit to action that aligns with their values. It emphasises the ubiquity of human suffering and recognises the importance of living by one’s values. Thus, it aims to help those with “actual, uncontrollable, deeply painful life experiences, rather than those whose suffering is with distorted thinking” (Trunzo, 2019). ACT helps by increasing “psychological flexibility” (which is the ability to be aware and mindful of your present moment in a non-judgemental way), even when the individual is experiencing feelings of pain or discomfort which might ordinarily limit apparent options. By noticing such feelings and sensations, it allows people to make decisions that are not held hostage to these uncomfortable feelings but rather geared towards meaningful action. ACT encourages individuals to notice their discomfort and enhance psychological flexibility to promote a more effective and meaningful life, despite the painful thoughts and feelings that come with existence. In summary, ACT has six core processes: acceptance (opening up to difficult thoughts and feelings instead of resisting them), cognitive defusion (stepping back from unhelpful thoughts rather than being controlled by them), present-moment awareness (mindfully engaging with the here and now), self-as-context (seeing oneself as more than thoughts and emotions), values (identifying what truly matters), and committed action (taking consistent steps towards those values, even in the face of challenges). Together, these processes are thought to cultivate psychological flexibility, helping individuals live meaningfully despite difficulties (Hayes et al., 2011).

When considering the Islamic model of the self (Rothman & Coyle, 2018), these principles of ACT can be integrated at the level of the *‘aql* and *nafs* to produce positive behavioural actions, like that seen in CBT. Where ACT goes further is that it intersects with an Islamic conception of suffering, acceptance, and meaning. In ACT, one is encouraged to live with uncomfortable thoughts and ultimately change one’s relationship to them. This supports the aim to achieve submission and surrender with *Allah*, where *Allah* is the ultimate destination despite the struggles that come with living in the ephemeral world. In ACT, there is a clear pathway to turn to the transcendent which in the Islamic paradigm is the source of all healing while living one’s life. In the words of Stephen Hayes regarding ACT, this means submitting and relying on a connection to something bigger than oneself in order to fully be present to what matters: “we’re going to feel what is there to be felt even though its hard….it’s about connecting to that spiritual side of you and from there directing your attention flexibly, fluidly, and voluntarily towards what is there to be focused on” (Hayes, 2016).

## Mapping ACT Processes onto the Islamic Self

We can identify the processes of ACT that correspond to different aspects of the Islamic self. This is essential for using ACT as a framework that effectively engages with Islamic beliefs. By applying Rothman and Coyle’s (2018) model of the Islamic self—comprising *nafs*, *aql*, *qalb*, and *Ruh*—we can map these concepts onto the relevant ACT processes (Table 1). The description relating to the part of the self is based on the Rothman & Coyle’s, 2018 model and Al-Ghazali (Al-Ghazali & Skellie, 2010; Rothman & Coyle, 2018). While other studies have matched ACT processes with relevant Islamic teachings—mainly using ACT as the foundation and interjecting with relevant Islamic guidance (Bhatti-Ali, 2023; Tanhan, 2019), our approach focuses on a more holistic engagement with the entire Islamic self as a foundation. This mapping is elaborated on below and summarised in Table 1.

**Table 1:** Mapping processes of ACT onto the Islamic self

Islamic structure of the self (Rothman & Coyle, 2018)	Description (Al-Ghazali & Skellie, 2010; Al-Ghazali & Winter, 2016; Rothman & Coyle, 2018)	Corresponding ACT process
*Nafs*	• Lower self—relates to behaviour, motivation, and impulse (means an aspect of the soul i.e. self or lower self)	• Committed action—to commit to goals that are set
	• Behavioural inclinations	
*Aql*	• Cognitive processing/Intellect/reason	• Identifying valued based goals and work towards them
	• Seen as a regulating factor to rein in inclinations from the *nafs*	• Mindfulness and non-judgemental approaches to observe one's behaviour and thoughts, and to defuse from them where appropriate
	• Cognitive awareness to connect with *Allah* who is transcendent	• Self as context
	• *Tawakkal* and *sabr*	
	• Can be an entry point to the spiritual, and to accessing the *Qalb* through exploring the relationship between *Aql* and emotion	
*Qalb*	• Spiritual heart	• Mindfulness of what is present in the here and now
	**•** Centre of emotion and spiritual awareness	• Connecting to the present moment
	• Leads towards or away from *Allah*	• Ubiquity of human suffering/acceptance
	• Impediments block connection with the Divine	
	• Requirement of self-reflection and cleansing	
	• Emphasises meaning in suffering and valued decisions	
*Ruh*	• Soul or spirit—the Divine breath or spark	• Acceptance and connection to which that extends beyond the self
	• Submission/acceptance	• Working towards values
	• Spiritual remembrance/worship	• Intention

### *The Nafs and the ACT Process of ‘committed action’*

Often translated as the self or ego, the *nafs* represents the base desires and appetites of the individual. In the Islamic tradition, it is associated with lower impulses and instincts, and it can either be in a state of spiritual purification or moral corruption. The purification of the *nafs* is central to the development of moral and spiritual character. ACT can engage with this aspect through committed action—committing to values-based steps to create meaningful action, even in the presence of difficult thoughts and emotions (Hayes et al., 2011). Committed action, in the context of controlling the *nafs*, involves consistently making choices that align with one's spiritual values, even when faced with inner temptations or struggles. For Muslim patients, this might involve persisting in acts of worship, self-discipline, and moral behaviour, despite the discomfort or resistance from the lower self. Rather than being ruled by fleeting desires, committed action calls for intentional steps towards self-improvement, patience, and a life grounded in faith.

Committed action also corresponds to the Islamic principle of intention (*niyyah*) and the concept of sin. It is not uncommon for individuals to relapse into old habits and give in to the demands of the *nafs*, which can undermine committed action. The Islamic narrative describes demonic whispering or the influence of the *nafs* as distractions from clear goals, with the devil (*Shaytan*) seeking to make the individual feel defeated and unmotivated if they ‘miss the mark’—that is, when they fall into sin thus failing to fulfil a religious obligation.

Intention (*niyyah*) is therefore crucial in this regard, as the Islamic tradition states: “Actions are judged by intentions, and every person will get what they intended” (Bukhari). This teaching provides hope and resilience in re-engaging with committed action if goals are not met.

### *The Aql’ and the ACT Processes of ‘self as context’, ‘defusion’, ‘mindfulness’, and ‘values’*

The *aql’* refers to the intellect or reason. It is the faculty responsible for rational thought, understanding, and decision-making. The ʿaql is understood as the part of the human being that can discern truth from falsehood and guide a person in making choices aligned with divine wisdom and ethical principles—comparable to the aspect of the self in ACT that identifies and commits to personal values. When an individual is fused with their thoughts, they often believe the critical appraisal they have of themselves and may be immobilised with anxiety over a stressful situation thus prevented from a useful action as a result. Individuals can become “hooked” on negative thoughts about themselves/situation. Defusion, a core process in ACT helps the individual put some space between the self and their thoughts. This enables them to better determine whether a particular thought matters to them or not, hence changing the relationship to the thoughts rather being held hostage by them. For Muslim patients, one could become forgetful of *Allah* in such situations, where the handing over to *Allah* while experiencing His Mercy or *Rahma* can facilitate the “untangling” from thoughts and of need to control them. Furthermore, by allowing the believer to connect with a larger body of awareness that offers hope and safety beyond the limits of the material world, the focus shifts to taking action despite uncomfortable thoughts (Tan, 2016). The Qur’an states: “God is enough for me: there is no god but Him; I put my trust in Him; He is the Lord of the Mighty Throne” (Haleem, 2005, p. 127). This verse describes submission to *Allah’s* utter Will (*tawakkul*) (Hamdy, 2009), and aids in the remembrance of being able to act in spite of the uncomfortable thoughts. It also mirrors the well-known prophetic tradition of tying ones camel (to prevent it from being stolen), then trusting in *Allah* i.e. do what is in your control, and leave whatever outcome up to *Allah* (Tirmidhi). Leaving the outcome to Allah, whatever that might be involves patience (*Sabr*). *Sabr* is described as “steadfastness in the religious impulse, in opposition of the impulse of desire” (Ghazzali & Littlejohn, 2016, p. 21). The Qur’an offers a positive outcome to those who do possess *sabr*: “…and those who persevere patiently will be given a full and unstinting reward” (Haleem, 2005, p. 296). It was the response from Prophet Jacob in the Qur’anic narrative when his sons falsely claimed that Jospeh had been eaten by a wolf. Though overcome with grief and anger, Jacob responded with profound patience and trust in Allah: “But it is best to be patient: from God alone I seek help to bear what you are saying” (Haleem, 2005, p. 146). From starting as a perception centred in the ‘*aql*, it can bridge to deeper experiences of submission and connection to *Allah* centred in the *qalb* or *Ruh.*

Aiding the defusion process is mindfulness. Mindfulness can be described as a state in which an individual becomes aware of the present moment, and non-judgementally observes rather than wrestles with thoughts, feelings and sensations (Goleman & Davidson, 2017). In ACT, the individual is invited to engage with the here and now through mindfulness. There has been an increase in the number of mindfulness-based therapies in recent years due to strong evidence of its effectiveness in treating psychological disorders (Hooper & Larsson, 2015). The most widely practised forms of mindfulness meditation in the western world are derived from Buddhist and Hindu traditions, where the individual pays close attention to their surroundings or their breathing (Goleman & Davidson, 2017). In the Islamic spiritual tradition (commonly known as ‘Sufism’ or ‘*tasawwuf*’), *dhikr* (literally translated as ‘remembrance’) is regarded as a mindfulness-based spiritual exercise centred on bringing the Divine presence into one’s consciousness (after lack of remembrance) and tempering the ego. Many spiritual masters would prescribe *dhikr* in the form of orisons^1^ to be recited consistently. Here, one is encouraged to be in a state of full presence, by establishing a point of focus in the mind—for example, envisioning the name of *Allah* written, or imagining the face of their spiritual master to aid concentration and channel spiritual energy. Any mental images or distracting thoughts that arise are either set aside or simply observed. *The Fragrant Scent*, the author, al-Aydarus, emphasises the importance of being aware of one’s inner thoughts to discern their origins (Al-Aydarus & Guezzou, 2016). He argues that understanding these motivating thoughts and their sources is essential, as sound actions in the sight of *Allah* require a pure intention. The “whisperings” of *Shaytan* (Satan) can be perceived as intrusive thoughts, requiring vigilance and a state of mental observance. This awareness helps distance oneself from unhelpful or distracting thoughts, enabling greater focus in prayer and fostering a deeper state of presence with *Allah*, the opposite of which is forgetfulness.

Although mindful observances are associated here as part of the ‘*aql*, deep awareness and connection cross over into the realm of the *qalb* especially when considering centring in the chest area and integrating the individual's felt sense.

### *The Qalb, and the ACT Processes of ‘present-moment awareness’, ‘mindfulness’, and ‘acceptance’*

Translated as the heart, the *qalb* is considered the seat of emotion, feeling, and spiritual awareness. In Islamic thought, the *qalb* is more than just a physical organ; it is the centre of a person’s consciousness and connection with the Divine. A pure *qalb* reflects sincerity, compassion, and closeness to *Allah*, while an impure one can be distracted or hardened by worldly concerns. It plays a crucial role in a person's spiritual journey, as it can either lead them towards *Allah* or be clouded by desires and distractions (Al-Ghazali & Winter, 2016). In Islamic thought, there can be impedimentsthat block the *qalb′s* connection to the Divine. These include being distant from perceiving the deeper realities of existence. In the Islamic spiritual tradition, the goal is to purify the *qalb* through devotional practices and reflection on oneself. An example articulated by Ibn Ata’illah al-Iskandari in his most famous work *Kitab al-Hikam* (Aphorisms) (Al-Iskandari & Danner, 2014). These aphorisms or wisdoms describe a practical guide towards purification of the *qalb*, and an embodiment of the virtues in Islam. For example: “Among the attributes of your human nature, draw away from every one that is incompatible with your servanthood, so that you may be responsive to the call of God (*Allah*) and near His Presence” (Al-Iskandari & Danner, 2014, p. 151).

This aphorism speaks to the individual who carries a sense of unworthiness, which then becomes a barrier to faith and connection. According to the spiritual tradition associated with Al-Ghazali, the key to overcoming this crisis is deep, honest self-reflection. One must sincerely examine what is causing these feelings of unworthiness. Thus, true healing comes from confronting oneself internally. Through this process, one realises that what truly resides within is *Allah*, and it is the unresolved flaws and sins that an individual may want to address—that obstructs Divine presence (Al-Ghazali & Skellie, 2010; Al-Ghazali & Winter, 2016). The Qur’an recognises that the process towards transformation and Divine presence is one that can cause pain and suffering. The Qur’an states:

We shall certainly test you with fear and hunger, and loss of property, lives, and crops. But [Prophet], give good news to those who are steadfast - those who say, when afflicted with a calamity, ‘We belong to God and to Him we shall return’. These will be given blessings and mercy from their Lord, and it is they who are rightly guided (Haleem, 2005, pp. 17–18).

A spiritually integrated ACT approach offers Muslim patients with a philosophy to exist beyond worldly suffering, comitting to value-based decisions, and finding meaning within suffering rather than avoiding it.

### *The Ruh, and the ACT Processes of ‘acceptance’, ‘present-moment awareness’, and ‘values’*

The *Ruh* is the soul or spirit—the Divine breath or spark that gives life to the body. In Al-Ghazali’s framework, it refers to the spiritual essence of a person. It is a divine, subtle substance that originates from *Allah* and is the source of life and consciousness (Al-Ghazali & Skellie, 2010). When combined with the body, it gives rise to the *nafs* (self), which has both higher, spiritual inclinations and lower, animalistic desires. In some contexts, *Ruh* is also used to translate Aristotle’s concept of *pneuma*, meaning the vital life force that spreads throughout the body. However, in Islamic thought, it retains a sacred dimension, connecting the human being to the divine realm. It is the eternal aspect of the human being directly connected to *Allah*, and it represents the potential for spiritual elevation and closeness to Him.

There is a relationship between acceptance in the face of adversity that can break down psychological attachments, allowing a deeper and more expansive connection beyond the self. Taylor (2021) highlights how suffering—especially when faced with acceptance rather than avoidance—can break rigid attachments to the self and open individuals to spiritual transformation (Taylor, 2021). In ACT, this process can be described as psychological flexibility, where individuals learn to let go of attachment to distressing thoughts and emotions, choosing instead to focus on a values-based living. Similarly, in Islamic spirituality, submission to Divine Will (*tawakkul*) is central to navigating hardship, as it allows an individual to detach from ego-driven suffering and embrace a higher, divine purpose. One of the qualities that link psychological flexibility to a core spiritual goal in Islam is transcendence (Pargament, 2011). Transcendence in Islam refers to the nature of *Allah* being beyond the material, while in ACT, being present in difficulty without attachment to the ego fosters a sense of connection to something greater than oneself.

While the *qalb* plays a central role in one’s spiritual awareness and emotions, it is ultimately the *Ruh* that facilitates transcendence—a shift beyond worldly distractions towards divine connection. In Islamic thought, suffering can cloud the *qalb*, thus acting as a barrier to divine perception. However, through acceptance, self-reflection, and devotion, these spiritual obstacles can be overcome, allowing for divine connection through the *Ruh*. Similarly, psychological flexibility in ACT encourages individuals to engage with suffering rather than avoid it, recognising that pain itself can be a path to transformation. The Qur’an states: “There is nothing like Him: He is the All Hearing, the All Seeing. The keys of the heavens and earth are His; He provides abundantly or sparingly for whoever He wills; He has full knowledge of all things” (Haleem, 2005, p. 29).

Such verses invoke a profound awareness of something greater than the self that purposefully determines all outcomes, resonating with ACT’s principle that true well-being does not come from controlling thoughts and emotions, but from acceptance, openness, and commitment to higher values. A spiritually integrated ACT approach offers Muslim patients a framework for navigating suffering—not through avoidance, but through meaning-making and surrender—a principle deeply rooted in the Islamic tradition.

## A Case Study

As part of the Survivors Rehabilitation Evaluation after Cancer (SURECAN) trial investigating if ACT improves the quality of life of cancer survivors, the study team interviewed participants on the trial who received the ACT intervention (Donovan et al., 2024). The SURECAN intervention aims to be culturally acceptable to diverse groups. Therefore, authors IK and DR suggested the research team asked participants who professed a spiritual or religious belief, how well ACT aligned with their beliefs. Out of the 14 participants interviewed, one self-identified as Muslim. For this case study, we examined the data collected about the participant’s spirituality to investigate the scope of experiences that related to ACT. In addition, how these experiences engage with parts of the Islamic self.

The participant is a middle-aged woman, who self describes as Muslim and originally comes from a South Asian country. The participant is in remission from breast cancer, and part of her treatment involved a mastectomy. The research used a semi-structured approach to interviewing, using a topic guide to focus on experiences and perceptions about receiving ACT therapy. Included in the topic guide was a question specifically probing the acceptability of ACT considering religious beliefs—“What way if any did ACT+ fit or not fit with your beliefs?” The data collected around this question during the interview were analysed using the framework shown in Table 1.

The participant highlighted that ACT did not conflict with their religious beliefs but rather encouraged them to draw strength and support from their spirituality. For example, in the below quote from the interview, the participant shared that the therapist delivering ACT encouraged the participant to recognise and utilise their religion as a source of comfort and resilience during challenging times:

Because she said to hold on and not to problem the religion, not to question it, nothing to do with religion, but actually just… So if you find strength from that, if you find comfort from that, then go back and who can help you do that? So, it helped me recognise that actually ACT pushed me back, not to question why me, but actually to say what can I do? Maybe what could support me? What is there in the religion that I could hang onto and support and move this forward? And I think that helped.

The participant did not identify any conflict with their religious beliefs and ACT—in fact, the ACT practitioner encouraged them to look deeper at the resources of their faith to support themselves.

Recognising and leveraging these values engages with the *aql* part of the self. This encouragement to proactive action is indicative of goal setting where progress towards a specific goal i.e. talking to the right person, may be achieved (or not). This aligns with the ‘*aql* but also addressing the *nafs*—especially if the therapist monitors progress towards achieving that goal which will leave room for the *nafs *not wanting to be challenged. The participant recognises it’s a valuable thing to do but may need help to challenge the *nafs *through therapist encouragement and monitoring towards carrying it out.

…she tapped into my interests, like my spirituality, my religion, and she helped me find the people in my…and look for the people who I could talk to, who would understand me. So I think even though she didn't match my culture or my religious background she helped me use that more proactively than I was, and in a positive way than I was… And then she asked me to think about the people in my life who I could get support and guidance and would help me. And it just so happened the Imam of the mosque, he's a family friend. He's rang about something else and I took that opportunity, followed by that session to ask him, I said I think I'm struggling, I need some help, some guidance. And he was able to send me some prayers and then check-in with me. And I don't know if I'd done that had I not had that…why wouldn't you ask him? That's what he's there for, he's there to support you. And again, it was a person of trust I could turn to. So yeah, for me, yes, I'm a spiritual and religious person, and ACT didn't detract from that at all. It supported.

In addition to being encouraged to identify and seek support based on their values, the participant expressed satisfaction with the therapist—who appeared not to share the same religious beliefs—for their proactive efforts to engage with the participant’s faith perspective. It is not clear if all ACT therapists would work in this way. However, in the context of the probes asked by the interviewer, there is a focus on the therapist being able to recognise and work with the participant’s religious beliefs. More data are required to assess how well—and in what depth—ACT therapists engaged with the participants’ expressions of faith. For example, the participant mentioned she received prayers from the Imam, therefore it may be beneficial to explore what kind of prayers, and what were their meaning. This might be a way to allow the participant to further explore the connection to the Divine, whilst forming trust and acceptance in the therapeutic relationship. The case study, however, indicates promise in a faith integrated intervention being delivered regardless of the therapist’s faith (or lack of). The participant also spoke of “Gods plan” (below) which suggest an intention to find acceptance of their circumstances, while continuing to work towards finding support from religion. The participant comments:

I'm quite a religious and spiritual person, but anything like this kind of…you asked the question, why me? And I believe…and religion for me, it was like God's plan…that kind of thing. But I think it didn't jar from ACT, because she made it…she made me look at what strengthens you, where could you get the support? Could you… And it was like where is the strength coming from within your own experience? And for me religion was one of those.

The participant mentioned that this form of “God’s plan” did not “jar with ACT” which suggests that ACT can integrate well with the cognitive acceptance understood by Muslims relating to submission to God’s (*Allah’s*) plan. Considering our framework and the Islamic self, this relates to the ‘*aql* and potentially *qalb* aspects of the self. Further exploration related to the degree of acceptance is required to fully address the *qalb*.

Overall, we can see from the small amount of data examined around this case study, that there is potential for ACT to work with a Muslim’s beliefs, and that the processes of ACT can address parts of the Islamic self. Further work needs to be carried out to fully explore how acceptable ACT would be for Muslims wanting toinclude faith in their therapy, and if it has the potential to address the components of the Islamic self.

## Discussion and Further Research

In this paper, we have highlighted the need for a tailored mental health intervention, with a broad conceptualisation of well-being, for Muslim patients as an under-served group. Due to Muslim patients having specific ways in which their beliefs serve as a source of support (or not), there is a need for a therapeutic approach that works with these beliefs, rather than ignoring them. An approach drawing from the Islamic tradition can potentially address the need for Muslims to have their beliefs more actively engaged with in the therapeutic encounter.

We have shown in this paper, using the Islamic conception of the self-developed by Rothman and Coyle (2018), that acceptance and commitment therapy (ACT) has the potential to be a good fit within an Islamic therapeutic paradigm, as there is alignment between the conceptualisation of suffering and the therapeutic processes involved. ACT can, therefore, provide a framework and language that allows for the integration of Islamic perspectives within therapy that could be delivered irrespective of faith background of the therapist. This has been partially demonstrated through the case study, but due to data limitations (e.g. *n*=1), much more work is needed.

While the processes of ACT align well with Islamic approaches to suffering and well-being, the underlying theoretical basis of ACT does not fully correspond with Islamic Psychology. ACT is rooted in functional contextualism (i.e. understanding the function of behaviour in the context in which it happens, so we can focus on what works, rather than right and wrong) and Relational Frame Theory (i.e. how language can entangle us in unhelpful thinking to create suffering), suggesting that human suffering arises, in part, from cognitive processes that are mistakenly taken as truths (Boone et al., 2015). The philosophical stance in ACT maintains that psychological suffering results from attempts to control or avoid distressing thoughts, emotions, and sensations, rather than accepting them as natural aspects of human experience. While this bears similarities to Islamic notions of surrender and acceptance, the point of departure remains clear: Islamic Psychology situates the source of human experience in Divine Will, whereas ACT is designed to operate within a secular, behavioural framework.

Despite this theoretical divergence, ACT remains a valuable framework for Muslim patients, particularly because its processes support alignment with an Islamic perspective on suffering and personal growth. It facilitates value-driven action, allowing individuals to respond to distress in a way that aligns with their faith, rather than being governed by experiential avoidance. Thus, while ACT may not fully integrate with the ontological foundations of Islamic Psychology, it offers a practical and flexible structure that can support Muslim patients in living in accordance with their values, and it may potentially make links to deeper experiences of the Divine Will. The acceptance and commitment components of ACT provide a clear direction for how Muslims may navigate suffering through faith.

Traditional Islam defines the self through spiritual dimensions, such as the soul (*Ruh*) and divine connection, while ACT is patient-focused, values-driven, and highly respectful of autonomy. This raises the challenge of balancing a framework grounded in Islamic teachings with the flexibility needed to respect personal interpretations of faith. While many Muslims seek therapy that incorporates their beliefs, there is variation in how individuals relate to Islamic concepts, creating potential tension between a structured theological model of the self and the diverse ways Muslims experience and practise their faith. The acceptability of this approach must therefore be explored further before developing such an integrated intervention. We have been successful in obtaining an NIHR grant to explore the acceptability of ACT among Muslims facing psychological distress or a long term health condition. The aim is to develop better guidance for Muslim patients and therapists on integrating faith within therapy. This initiative seeks to bridge ACT with Islamic teachings in a practical manner, allowing therapists (irrespective of faith background) to use ACT as a scaffold for Islamic integration. Practically, this would enable therapists to guide individuals towards reframing emotional responses in a healthier way—surface-level adjustments that remain consistent with Islamic values. At a deeper level, ACT can support Islamic notions of self-examination and surrender, allowing therapy to facilitate honest reflection on an individual's life, aspirations, and spiritual connection. Subsequently, further research is needed to pilot an intervention, develop practical applications for therapists, and assess effectiveness in improving mental health outcomes. We will also need to evaluate accessibility and ensure that such an approach effectively supports a diverse Muslim community.

## Conclusion

This paper highlights the need for mental health interventions that addresses some of the barriers associated with poorer service use and outcomes among Muslims. Religious beliefs less often feature as an included dimension in intervention development and can potentially have a broader effect than culturally specific interventions. Acceptance and commitment therapy (ACT) although not fully aligning with the theoretical foundations of Islamic Psychology offers a valuable framework for working with Muslim patients to integrate holistically with the Islamic self. Further research is necessary to evaluate the effectiveness and acceptability among Muslims of this approach. Future efforts aim to develop guidance for integrating Islamic principles in therapy, ultimately enhancing mental health outcomes for Muslim patients facing adversity.

## Footnotes

1 Orisons are intimate discourses with God. It has been used by Shakespeare in ‘Romeo and Juliette:’ *“…I pray thee, leave me to myself to-night, For I have need of many orisons****,**** To move the heavens to smile upon my state, Which, well thou know’st, is cross, and full of sin…” (The Oxford Shakespeare: Romeo and Juliet IV:3).* It is a close approximation to the Arabic word *awraad*.
